# Research on potential disruptive technology identification based on technology network

**DOI:** 10.1371/journal.pone.0298098

**Published:** 2024-04-04

**Authors:** Mingli Ding, Wangke Yu, Ran Li, Zhenzhen Wang, Jianing Li

**Affiliations:** 1 Intellectual Property Information Services Center, Jingdezhen Ceramic University, Jingdezhen, China; 2 School of Information Engineering, Jingdezhen Ceramic University, Jingdezhen, China; Polytechnic Institute of Portalegre: Instituto Politecnico de Portalegre, PORTUGAL

## Abstract

Three evident and meaningful characteristics of disruptive technology are the zeroing effect that causes sustaining technology useless for its remarkable and unprecedented progress, reshaping the landscape of technology and economy, and leading the future mainstream of technology system, all of which have profound impacts and positive influences. The identification of disruptive technology is a universally difficult task. Therefore, this paper aims to enhance the technical relevance of potential disruptive technology identification results and improve the granularity and effectiveness of potential disruptive technology identification topics. According to the life cycle theory, dividing the time stage, then constructing and analyzing the dynamic of technology networks to identify potential disruptive technology. Thereby, using the Latent Dirichlet Allocation (LDA) topic model further to clarify the topic content of potential disruptive technologies. This paper takes the large civil unmanned aerial vehicles (UAVs) as an example to prove the feasibility and effectiveness of the model. The results show that the potential disruptive technology in this field is the data acquisition, main equipment, and ground platform intelligence.

## 1. Introduction

Disruptive technology has a high value in technology strategies and investment decisions [1]. As a great development and growing route of traditional technologies, disruptive technology plays an important role in advancing human society, high-quality economic progress, and improving living conditions and national security. The report of the 19th National Congress of the Communist Party of China put forward corresponding requirements, focusing on key common technologies, frontier-leading technologies, modern engineering technologies, and disruptive technological innovation [2]. The " 13th Five-Year National Science and Technology Innovation Plan " and the " National Innovation-Driven Development Strategy Outline " included disruptive technological innovation. Beijing municipal party committee and municipal government attached great importance to constructing science and technology innovation. The moon landing plan proposed by Japan, the Russian Forward-looking Research Foundation, the British Advanced Research and Invention Agency, and the US Defense Advanced Research Projects Agency. Many countries and regions, government departments, and large enterprises have participated in the party to promote disruptive technological innovation [3].

However, disruptive technology in most cases is not easy to judge or identify, because of its long brewing and germination process. Thus, compared to existing sustaining technologies, it has fallen behind in core indicators from the early stage. Still, for its significant and rigid advantages in certain non-core performance indexes, disruptive technology can exist for a long time and not be replaced by any other technologies. Once, it enters the development period, the field will promote sound and rapidly. Therefore, disruptive technology identification is a necessary research task.

## 2. Literature review

’Disruptive technology’ [4] is a kind of new and imaginative technology, which has a disruptive effect on existing traditional or mainstream technologies. At present, there are three main methods concerning disruptive technology identification: technology roadmap and expert experience combined analysis, index evaluation analysis, and model-based analysis [5]. Among them, Vojak and Chambers (2004) [6], Kostoff (2004) [7], and Walsh (2004) [8] combined analysis of technology roadmap and expert experience towards disruptive technology identification based on the development or improvement of the technology roadmap. Carlsen (2010) [9], Lu Guangsong and Lu Ping (2011) [10] discussed and evaluated the effect of technology roadmap analysis in the identification of potentially disruptive technologies. Based on the index evaluation analysis method, Sainio and Puumalainen (2007) [11], Keller and Hüsig (2009) [12], Ganguly (2010) [13], Hang (2011) [14], Hardman et al. (2013) [15], Jia Weifeng et al. (2022) [16], and Chen Xiaoli et al. (2019) [17] put forward the corresponding subversive technical index standard theory. In terms of model analysis, Lim and Anderson (2016) [18], Adner (2002) [19], Su Qilin (2006) [20], Linton (2002) [21], A. Momenni (2016) [22], and Jia Weifeng et al. (2021) [23] established corresponding models to explore the identification of disruptive technologies.

In detail, Ganguly has implemented a potential disruptive technology evaluation system, and developed indicators of technology adoption rate, target market segmentation, technology expected utility, and existing technology maturity. Taking the online music industry as an example, it compares the cost per channel, flexibility, acquisition speed, accessibility, and robustness with existing technologies. However, there are still some limitations in the evaluation system of some indicators that still need to be combined with more qualitative recognition, music quality portability, functionality (feature + performance), and analysis. Moreover, the defined indicators lack versatility and must be customized and adjusted to apply to different industries or products. Lu Songguang and Lu Ping formulated a technology roadmap to identify the potential disruptive technologies, then pointed out the problems of stakeholder participation, technology uncertainty, market uncertainty, infrastructure uncertainty, path dependence, and path creation in this method. A.Momenni advocated identifying potential disruptive technologies through topic modeling based on k-core analysis, patent development paths, and past and current trends in technology development. This study only analyzed some invention patents, and there was information loss in the analysis process. In addition, due to the limitations of the algorithm, the number of patents analysis is also limited. At the same time, there are still some shortcomings, such as the technical classification is not clear and comprehensive, and the number of citations between different patent offices is different. From this point of view, there is still some research value in the identification method of disruptive technology.

Considering the above problems, below will explore to identify disruptive technology with patent data in the technology network. The patent literature is a major carrier of scientific and technological information. It has always been the object of analysis that researchers strived to mine and analyze disruptive technologies from it. In recent years, researchers have relied on patent data to continue to explore the direction of disruptive prediction through data mining, and have successively provided a number of innovative solutions. In the past, the analysis of disruptive technology co-occurrence networks mainly focused on the citation network, which stressed the classification of high-value patents, and had little direct correlation with technology, but the International Patent Classification (IPC) codes were closely related to technology. In the meantime, the LDA topic model was also used to explain and refine the results, to improve the accuracy and possibility of disruptive technology identification.

## 3. Research design

As an important indicator and carrier for measuring technology, patents contain more than 90% of the world’s technical information [24]. Considering the diffusion and transition of disruptive technological innovation, the hierarchical analysis method is used to realize technology identification from three levels: knowledge flow, invention patent, and technology theme. First, collecting the technical development data of the whole large-scale civil UAVs field, dividing the life cycle according to the trend of patent application, and grasping the development trend of this field. With the accumulation of basic knowledge theory in the early stage, technology gradually awakens and develops to generate invention patents and transition diffusion. Through the analysis of the network at the end of the germination period and the early stage of the development period, could measure the changes and transitions of each node. The rapid development and application diffusion of invention patents will continue for a long time, and many broad invention patents will gradually clarify and converge on the specific leading technologies.

The three different analyses lay the basis for identifying potential disruptive technologies and play an active role in scientific research and technology discovery. Knowledge Flow: This involves understanding existing theoretical knowledge, analyzing the technological life cycle in the field to gain in-depth learning, and applying knowledge practically. This approach facilitates knowledge reproduction and co-production. Invention Patents: These patents encompass both existing scientific and technological achievements as well as the latest breakthroughs, reflecting the pulse and direction of technological development. They serve as a crucial entry point for discovering technological innovation points and emerging solutions. Technological Themes: With continuous development, rapid growth, and integration of related technologies, these themes can be transformed into actual productivity and contribute to the sustainability of patent value. Through this method, we can identify potential disruptive technology themes from a vast expanse of knowledge. Furthermore, this provides a reference for enterprise technological research and development, as well as the positive development of the industry.

Mining the contained meaning in the feature items and the content of the information, and quantifying the information analysis carrier of the technology network. IPC represents the relationship and strength between specific technical topics. The influence of IPC cannot be measured individually. Only in the co-occurrence network can show its influence in the process of technical cooperation or technology absorption and diffusion. Therefore, taking advantage of IPC to construct a technology co-occurrence network for analysis and using its correlation degree and co-occurrence frequency to mine potential disruptive technology. Based on the theory of co-occurrence network and previous studies, the information of each node in the technology network is measured by four indicators: degree centrality, closeness centrality, betweenness centrality, and eccentricity.

The influence of IPC is determined by their control ability in the technology network and their dependence on other IPC codes, see Eqs (1) to (4). Degree centrality can calculate the degree to which a node is associated with all other nodes in the network and is the simplest index to quantify the influence of nodes. The larger the degree of a node is, the higher the degree centrality of the node is, which means that the node is more important in the network and the cohesion is stronger. The betweenness centrality depends on the degree to which a node is at the center of multiple nodes, which can be measured according to the mediating role of nodes in the network. The high betweenness centrality of the node means it is located on the shortest path of multiple other nodes, which indicates that the node has strong technical or resource control ability in the technology network. Closeness centrality can be used to check whether the node is at the core of the technology network, intending to the closeness of the node to all other nodes in the network. The eccentricity implies the distance from a given starting node to the farthest node from it.


$$DC_{i}=\frac{1}{N-1}\sum_{j=1}^{N}k_{ij}(i\neq j)$$ (1)

$$BC_{i}=\frac{1}{(N-1)(N-2)/2}\sum_{i\neq j\neq t}\frac{g_{jt}^{i}}{g_{it}}$$ (2)

$$CC_{i}=\frac{N-1}{\sum_{j=1}^{N}d_{ij}}$$ (3)

$$EC_{i}=\max\{d_{ij},\forall j\in N\}$$ (4)

The Eq (1) is the calculation formula of the degree centrality of each node, represents the connection relationship between node i and node j, and N represents the total number of network nodes; the Eq (2) is the calculation formula of the betweenness centrality of each node, represents the number of shortest paths that node j and node t are connected and pass through i, represents the number of all shortest paths, and N represents the total number of network nodes. Eq (3) is the calculation formula of closeness centrality, represents the network distance between node i and node j, and N is the total number of network nodes. The Eq (4) is the eccentricity calculation formula, is defined as the same Eq (3), and N is the total number of network nodes. In the germination stage, according to the relevant indicators, the low-impact high-potential nodes are divided into two types: the nodes in type 1 have the characteristics of low degree centrality, low betweenness centrality, and high closeness centrality. After entering the technology network, they are connected with important nodes and closer to the center position; the nodes in type 2 have the characteristics of low degree centrality, low betweenness centrality, and high eccentricity. When entering the technology network, they are not connected to the important nodes of the existing network and are in a more remote network position.

**Fig 1:**
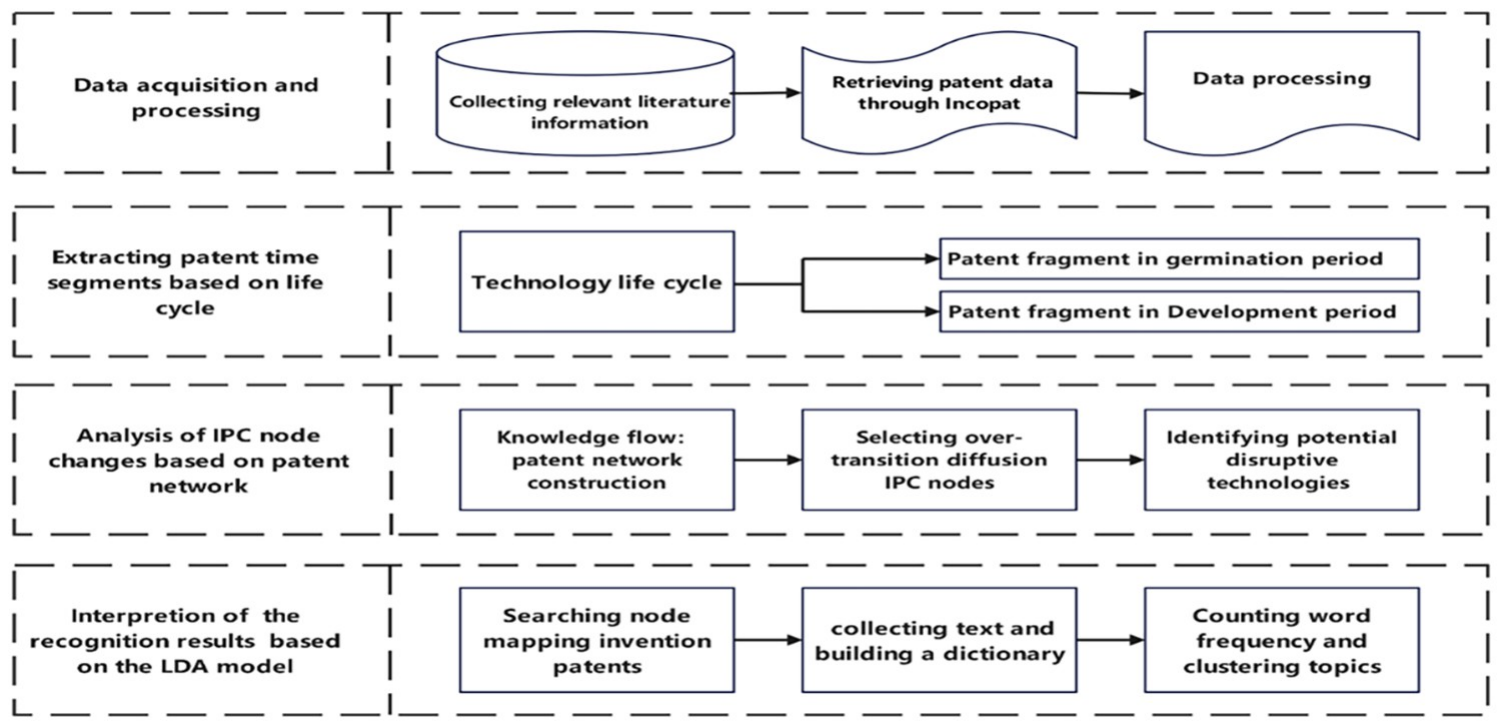
Potential disruptive technology identification flow chart.

In order to further analyze the potential disruptive technologies in the field, the LDA topic model is used to interpret the invention patent abstracts mapped by the transition diffusion IPC. On the basis of the identification of the transition and diffusion of the technology network nodes, a more fine-grained analysis is carried out to verify the results of the analysis obtained before, furthermore to determine and refine the specific research and development direction of the previous identification technology results. The overall analysis process is shown in Fig 1.

## 4. Empirical analysis

### 4.1 Data acquisition and processing

Firstly, Familiar with the development status of this field is preliminarily, and the technical decomposition of the required research field through the paper and patent query. Meanwhile, constructing the retrieval strategy, in consideration of the previous decomposition of the key technologies of large civil UAV patents [25]. Moreover, obtaining the relevant invention patent data through the Incopat patent database, and then preprocessing the data to facilitate the analysis of the subsequent technology network.

The UAVs have the advantages of low cost, small size, flexible maneuverability, convenience to use, strong combat survivability, and low combat environment requirements, which are valued by domestic and foreign research and development teams. After more than 30 years of accumulation and development of the domestic UAV application market, the demand scenario has gradually expanded from the initial military to consumption. According to the application field of UAVs, it can be roughly divided into military type and civil type. Large civil UAVs are one of the future development hotspots in the field of UAVs. With the continuous research and development of civil UAVs in various countries, it has been widely used in agricultural plant protection, film and television aerial photography, transportation, emergency search and rescue, power inspection, communication relay, meteorological monitoring, environmental protection, public safety, and other fields. Therefore, it is of great significance to identify the potential disruptive technology of large civil UAVs.

### 4.2 Life cycle analysis

Disruptive technology essentially belongs to technologies, which is in line with the general law of technological development. The evolution rules are consistent with the general law of the technology life cycle, and show different characteristics at different stages. Therefore, the technology life cycle is used to divide the development stage and show the dynamic development process of disruptive technology innovation. Due to the small number of patents in the field before 1970 and the slow development, the patent data from 1971 to 2020 are selected for patent life cycle analysis. According to the search results, this paper uses Loget Lab 4.0 software to carry out logistic curve fitting and divides the patent life cycle of a large civil UAV into four cycles, namely the germination period, growth period, maturity period, and decline period. Among them, the germination period is 1973–2012, the growth period is 2013–2016, the maturity period is 2017–2034, and the recession period is after 2035. According to the characteristics of slow development in its germination period and gradual expansion in its growth period, the whole germination period from 2003 to 2007 was selected as the first stage, from 2008 to 2012 as the second stage, from 2013 to 2015 as the third stage in the early growth period, and from 2016 to 2018 as the fourth stage. The technology network was constructed and the relevant index data were analyzed.

According to the change in the position of the technology nodes in the network in the germination stage and the growth stage, the IPC codes with subversive potential are selected.

### 4.3 Potential disruptive technology identification results

According to the technology network constructed in the germination stage, the indicators of each node are measured to determine the distribution of each node in the technology network. In the first phase, G05D1 / 00 (control of the position, course, altitude, or attitude of a vehicle on land, in water, in the air, or in space, such as an autopilot (a radio navigation system or a similar system using other waves to enter G01S) [2006.01]) occupies the core position of the network and represents the flight control technology of a large civil UAV. The flight control system is the key technology of application and development in the field of UAVs [26]. The flight control system is the key core part of UAV control. As the brain of a UAV, it has an irreplaceable position, and generally includes three parts: sensor, airborne computer, and servo action equipment. The functions realized mainly include three categories: UAV attitude stability and control, UAV mission equipment management and emergency control, as shown in Table 1.

**Table 1:** The calculation results of each IPC node location index.

IPC	degree centrality	Eccen-tricity	closeness centrality	betweenness centrality	description
H04B7/00	0.06	1	1.00	0.00	Radio transmission system
H04L29/00	0.04	2	0.71	0.00	Devices, equipment, circuits, and systems not included in individual groups H04L1/00 to H04L27/00
G01S5/00	0.04	2	0.71	0.00	Positioning by determining the fit of two or more direction, distance, or position lines
H04L12/00	0.03	2	0.63	0.00	Data exchange network
G08G1/00	0.01	1	1.00	0.00	Traffic control systems for road vehicles
---	---	---	---	---	---
F42B10/00	0.03	6	0.33	0.00	Effect devices, e.g. to improve the aerodynamic properties of the projectile or launcher, etc.
A62B1/00	0.01	6	0.28	0.00	Device for lowering a person from a building or similar object
A62C99/00	0.01	6	0.34	0.00	Other technical topics in firefighting.
G06K7/00	0.02	6	0.32	0.00	Method or apparatus for sensing a recording carrier
B60F5/00	0.01	6	0.30	0.00	Other vehicles capable of traveling on or in a different medium

Through calculation, it is found that the number of nodes in the germination stage conforming to the two types described in Table 1 is 148. Furthermore, according to the technical development procedures, the degree centrality and betweenness centrality of the above nodes in the technology network of the two stages of the growth period are measured to test whether one or more indicators exceed the average level of the current period, so as to determine the expansion progress and influence degree of the nodes. As a result, at least one of the 91 node influence indicators was selected to exceed the average level of the current period, that is, at least one expansion transition occurred during the growth period. Therefore, compared with other nodes, they have the possibility of subverting the existing knowledge structure and theoretical basis in the field. Further found that H04N7 / 00 (TV system (components into the H04N3 / 00, H04N5 / 00: for digital video signal encoding, decoding, compression or decompression method or device; the degree centrality and closeness centrality of H04N7 / 00 increase from 0.03 and 0.41 in the first stage to 0.41 and 0.6 in the fourth stage. At the same time, in the fourth stage, it has a high betweenness centrality, which controls and restricts the development of other technologies in the field. This shows that image communication technology has potential subversiveness in the field of large civil UAVs.

**Fig 2:**
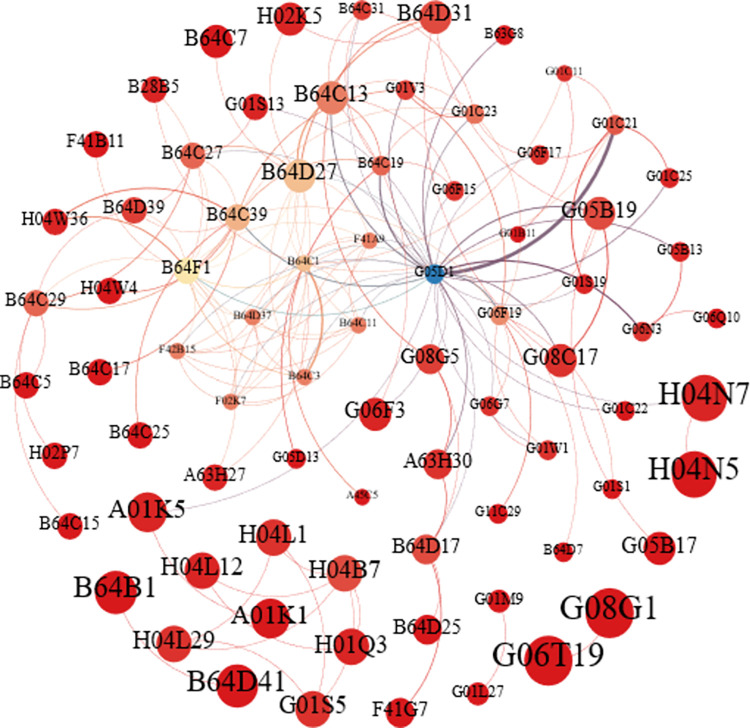
The first stage of the technology network.

In addition, from the overall observation of the technology network, it can be found that there is a possibility of important technological innovation in the field of large civil UAVs. Taking the first stage of the germination period as an example, its overall network presents a state where the center is more concentrated and the edge is scattered, and the IPC codes of the nodes with a higher degree of central technology integration is mostly concentrated on aircraft and regulation control. Distributed in the edge of the more dispersed nodes, such as the transmission of digital information; survey navigation; video metrology, or photogrammetry; measuring azimuth information such as level, distance or azimuth is also an important technology in the field of UAVs. It can be seen that in the early invention patent technology network, there are certain emerging technologies that disperse the edge of the network and have the possibility of diffusion and transition, as shown in Fig 2.

Finally, the IPC nodes of the transition diffusion in the knowledge flow are mapped to invention patents, and 1271 related invention patents are required to be analyzed specifically. There are several technical topics and research directions in this part of the invention patent, which need to be further focused and explained.

### 4.4 The interpretation and test of the LDA topic model

**Fig 3:**
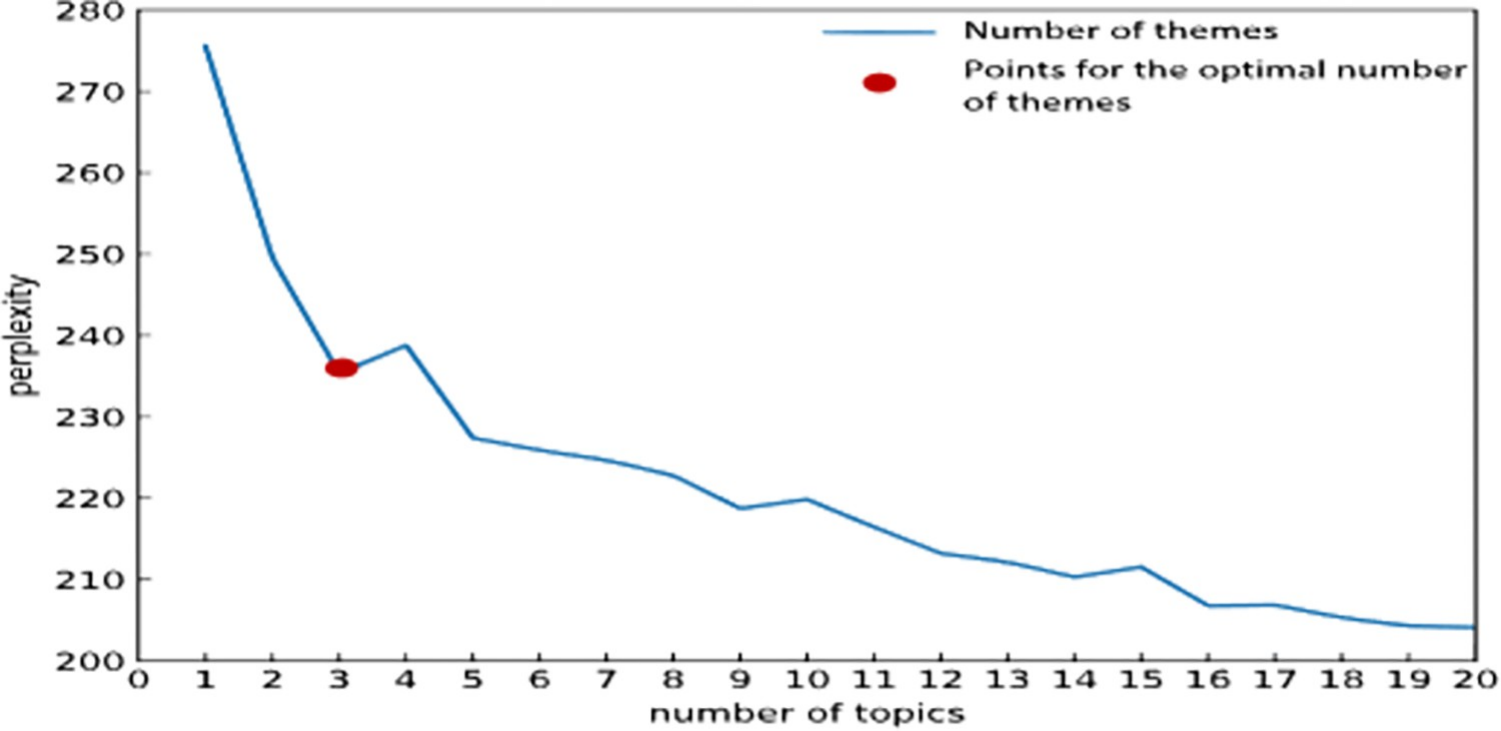
The number of topics perplexity curve.

Extract the abstracts of 1271 selected inventions, and combine the LDA topic model for technical topic clustering. The Python software Jieba and Gensim are used to segment words and remove stop words. The term frequency–inverse document frequency (TF-IDF) algorithm is used to calculate the frequency weight of technical keywords in the patent literature, and then the model is used to sort out the technical topics, and the perplexity value of each topic is calculated, as shown in Fig 3.

**Fig 4:**
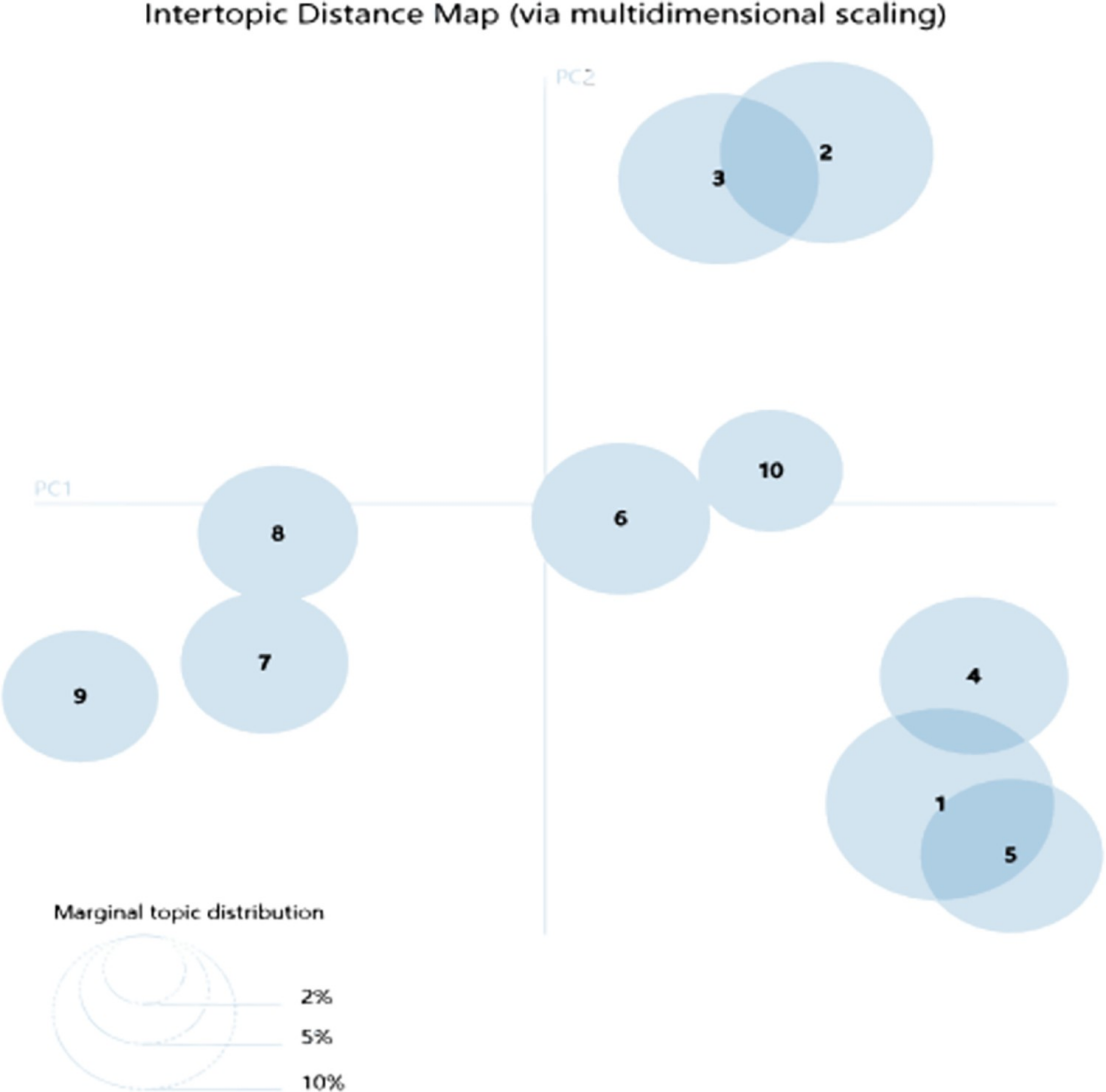
Intertopic distance map.

At the same time, as the intertopic distance map shows, according to the size and number of bubbles, found that the frequency of themes 1, 2, and 3 are higher. In addition, based on the analysis of the distribution dimension and location of the theme, noticed that theme 2 and theme 3 have the feature word intersection and close connection; compared with Topic 1, the Jensen-Shannon divergence (JSD) distance between the two topics is far, and the content is quite different, as shown in Fig 4.

Combined with the confusion curve and the actual analysis results, the optimal number of topics is finally selected to be 3, as shown in Table 2.

**Table 2:** Potential disruptive technology topics.

Theme number	Theme content and keywords
Theme 1	Data acquisition: target, image, terminal, information, method, aerial photography, object, vehicle, mobile, user
Theme 2	Main equipment: settings, ontology, aircraft fuselage, aircraft body, motor, rotor, controller, remote control, control station, propeller
Theme 3	Ground platform intelligence: detection, platform, ground, intelligent power, emergency, technology, patrol, personnel, line

Potential disruptive technology identification and interpretation of clustering topic results:

Theme 1 includes image equipment, information data, target area monitoring, and user terminal interaction are all associated with data acquisition, especially in the application of vehicle-mounted drones and autonomous driving. Further analysis of Theme 1 is provided below.

National deployment: in 2002, the U.S. Department of Transportation and the National Aeronautics and Space Administration focused on launching the application of UAVs in the field of transportation. In 2010, Shanghai purchased two small UAVs to participate in environmental resource management, maritime search and rescue, law enforcement investigation and evidence collection, traffic flow monitoring, and other work content, focusing on public emergencies and on-site emergency command of the information collection [27]. In the same year, China’s State Council and the Central Military Commission issued the Opinions on Deepening the Reform of China’s Low Altitude Airspace Management, which provided an opportunity for the promotion of drones in the civil sector, such as border monitoring, disaster monitoring, and assessment. Consumer drones are mainly used for leisure purposes such as aerial photography and gaming, and most of them adopt multi-rotor platforms with lower cost of construction. Enterprise R&D: In 2014, Renault released a KWID concept car, equipped with a quadcopter drone, which was usually automatically stowed on the roof of the car, and could be automatically flown and lifted into the air when needed through the built-in GPS and camera, combined with the tablet operation of the console. In 2015, Gopro, Inc. entered the "Small Drone Alliance" and disclosed several drones for use in aerial photography and gaming., and officially released the first multi-motor driven drone. Shenzhen DJI Innovation Technology Co., Ltd (DJI) also announced that it would apply self-developed cameras to its future drone products [28]. In early 2016, DJI released the Elf Phantom 4 drone, which was the first time it successfully introduced binocular vision technology in a drone, achieving autonomous following and autonomous obstacle avoidance, which was the key factor that prompted DJI’s decision to enter the field of intelligent driving. In 2017, Land Rover launched a production model of the vehicle drone, the Discovery SOV Edition. It was equipped with an onboard drone, which could take off and land the roof drone at high speeds on the car, through an electromagnetic holding and automatic positioning system. Industry development: Data Society and Digital Economy and the "Belt and Road" Cooperation International Academic Conference mentioned the role of information technology upgrades in the expansion of drone application scenarios. In 2019, the Ministry of Industry and Information Technology issued 5G licenses to China Telecom Corp Ltd, China Mobile Communications Corporation, China United Network Communication Group Co., Ltd, and other communication units, the construction of the network and the application of innovation go hand in hand [29]. In 2020, the civil drone industry launched the "Five-Year Action Programme for Promoting the Construction of a New Infrastructure for Civil Aviation", which proposed to expand the application scenarios of UAV logistics and transportation, terminal distribution, etc. At Some airports, it has achieved the related infrastructure of unmanned aerial vehicles, unmanned vehicles, and other operating conditions, to explore the military and civil aviation cooperation, manned aircraft unmanned aircraft fusion operation, and air-ground integration operation.

Theme 2 includes major airframe equipment such as airframe, motor, rotor, controller, remote control, controller, propeller, etc., involving airframe structure, electrical system, flight control system, and power system. The fuselage platform is the bearing platform of civil UAVs, involving the fuselage, arm, frame, landing gear, other structural components, and their connecting structures. The power system is the key guarantee for the endurance of civil UAVs, including piston, fuel gas, oil-electric hybrid, electric motors, and other modes. The aerodynamic layout is the vital assurance for the success of the flight of civil UAVs, and it can be classified into rotary wing, fixed wing, and mixed wing, the rotary wing can be specifically classified into single rotor, fixed wing, and hybrid wing [30]. The research and development direction of the UAV control system is closely related to the above theme, and the development of UAV technology and market demand [31]. The UAV control station is the command center where the operator can control and supervise the UAV remotely. At the same time, the control station is also equipped with a series of functions such as online mission planning, data control, image and intelligence processing, and view display [32]. One of the main reasons for civil UAVs to malfunction and go out of control is mechanical failure. In recent years, there have been several incidents at home and abroad in which drones crashed and hurt people due to battery or motor failures. The Karma drone launched by the foreign company GoPro, Inc. was also recalled because of the basic battery fixation problem. Therefore, the quality of the body structure, material, and manufacturing process of civil UAVs will directly affect the safety and operational efficiency of their operation. Without the influence of external factors, the internal stability of the UAV itself is also extremely important. Moreover, the quality of the materials, the compatibility of the internal structures, and the manufacturing process are all key elements that affect the safety and stability of UAVs [33].

Theme 3 indicates the detection and intelligence of the ground platform, especially in the application of emergency and patrol of electric power. The U.S. Federal Aviation Administration (FAA) proposed 19 types of services for Unmanned Aerial System Traffic Management. The European Single European Sky ATM Research (SESAR) listed 30 functions that U-space should have. The vast majority of the functions need to be newly defined and developed, and they were all based on digital and automated services for intelligent system function development. 2009 was the beginning year of inspection drones in China, with the State Grid formally setting up a project to develop a drone inspection system. 17 August 2021, San Diego—following the critical breakthrough of the Qualcomm Flight platform, which enabled the successful unmanned flight of the "Witty" helicopter on Mars. Thus, Qualcomm Technologies, Inc. continued to strengthen its leadership in the UAV space with the introduction of the world’s first 5G and AI-enabled drone platform, and the reference design of the Qualcomm Flight RB5 5G platform. In 2023, Foia Intelligent Technology Development (Jiangsu) Co., Ltd. (Fioa) launched the Zhifang A30EC series of UAV nests to become the ground infrastructure that enabled fully automated UAV operations. The compact and lightweight design paired with Fioa Intelligence’s RuiYun control system realized the automatic power change, battery management, temperature control and dehumidification, uninterruptible power supply, remote communication, intelligent lighting, and weather sensing. This innovative equipment completely changed the mode of manual on-site operation of UAVs, improved operational efficiency, and realized fully automatic operation of UAVs.

In summary, three potential disruptive technology directions are selected through technology network analysis and the LDA topic model. The future development trend of large civil UAVs is roughly the same as the identified technical topics, thus the identification method has certain effectiveness. Moreover, for the above technology direction, enterprises are active in R&D and innovation, patent applications are gradually increasing, technology development tends to be mature, and the market tends to be stable and saturated, thus it is more difficult to enter. The main patent applicants have rapid momentum of technology research and development, and the technology competition in the large civil UAV industry is more intense. It is recommended that they strengthen cooperation between industry, academia, and research to form a synergy in technology development and achieve breakthroughs in disruptive technologies.

### 4.5 Empirical test

To test the validity of the empirical data, use the spss26 software for the Mann-Whitney U test. First, the patents that do not have potential disruptive technologies in the germination period are set to 1 group and otherwise set to 2 groups. Secondly, the Citation Index and Inventor Index are selected as indicators, and the entropy weighting method and linear weighting method are adopted to calculate the corresponding index scores of patents. Among them, the Citation Index normalizes the patent age of each patent, which represents technological innovation and has a significant impact on subsequent technological development, the more often a patent is cited, the greater its patent value, see Eq (5). The Inventor Index measures the inventor of a patent, the greater the number of inventors on a patent, the greater the creative effort to develop the patents, see Eq (6).


$$Citation\;Index=\log_{10}\frac{Forward\;Citation\;Number}{Patent\;Age}$$ (5)

$$Inventor\;Index=\log_{10}(1+Number\;of\;Inventors)$$ (6)

After testing, the p-value was 0.034, lower than 0.05, the Z value was 2.118, the average rank of group 1 was 494.57, and the average rank of group 2 was 541.62. Therefore, the original hypothesis is rejected, and it is concluded that there is a significant difference between the two sets of data and the inventor in terms of the number of citations. In summary, the patents of the two groups are superior and subversive in terms of patent value, continuous influence, and creativity.

## 5. Conclusion

Main contributions and innovations: This paper simulates the development path of disruptive technology from knowledge literature, invention patents, and technology topics through an analytic hierarchy process to identify potential technology topics in the early stage of its development. At the same time, the technology network is constructed with the IPC codes of patents as the entry point, which enhances the degree of correlation between the recognition results and the technology compared with the citation network analysis. Meanwhile, in order to avoid the problem that the IPC codes range is relatively large, the LDA topic model is used to extract the technical topics that should be mapped to the invention patent, which improves the granularity and accuracy of the analysis results.

(1) According to the life cycle theory, the invention patent information from 2003 to 2018 is intercepted, and the technical network construction and dynamic analysis are carried out according to the invention patent IPC, and the node-image communication technology with prominent diffusion and significant transition characteristics is selected as the potential disruptive technology.

(2) Search for the invention patent information mapped by the potential disruptive IPC, and obtain the technical topic module of the data acquisition, main equipment, and ground platform intelligence through LDA topic model clustering. After querying and verifying the literature in the field, the topic identification results of the technology have a certain correlation with the development trend of the industry.

In view of the above analysis, there are still some shortcomings: this method only considers the IPC codes of patents and does not involve the technology market, consumers, policies, and other factors, so it has certain limitations. In addition, due to the time lag of patent data, the analysis method in this paper has limitations in the prediction of disruptive technologies. At the same time, it can be further combined with other literature, evaluation indicators, theme evolution analysis, and other comprehensive analyses and research.
